# German and Chinese dataset on attitudes regarding COVID-19 policies, perception of the crisis, and belief in conspiracy theories

**DOI:** 10.1016/j.dib.2020.106384

**Published:** 2020-10-14

**Authors:** Marc Oliver Rieger, Yanping He-Ulbricht

**Affiliations:** a Department IV, University of Trier, Research cluster “Cultures in Transitions in East Asia and Europe”, 54296 Trier, Germany; b University of Trier, 54296 Trier, Germany

**Keywords:** COVID-19 pandemic, Perceptions of government intervention, Government trust, Media trust, Conspiracy theories, social distancing, Face masks

## Abstract

This data article describes the attitudes of German and Chinese respondents to some measures taken against the COVID-19 pandemic such as social distancing and face masks wearing, as well as their trust in government actions. The data were collected through six online surveys conducted between March 23 to September 15 2020 from 865 participants in Germany, 135 in China and 169 participants with Chinese roots in Germany. The data were partly used in related research papers in which the theoretical background, analysis of the survey variables and the interpretation of the findings are presented in detail [1,2]. These survey data can be used in future studies of individual perception of the measures taken in the fight against the pandemic. The data cover topics which include, in particular, worries about the pandemic, estimations and expectations concerning the further development of the pandemic, perception of government responses and media coverage, attitudes towards social distancing and other countermeasures, and COVID-19-related conspiracy theories. Differences between Chinese and German respondents on some of these issues can also be studied with this dataset.

## Specifications Table

**Table:** 

Subject	Infectious Diseases
Specific subject area	Econometric models applied to infectious diseases’ epidemiological data to understand the worries about the pandemic, estimations and expectations concerning the further development of the pandemic, attitudes to countermeasures, factors contributing to different perceptions of government interventions and media coverage, and COVID-19-related conspiracy theories.
Type of data	Table
How data were acquired	Survey
Data format	Raw, filtered and partially analyzed. An Excel file with data is uploaded.
Parameters for data collection	No parameters
Description of data collection	The data were collected through five online surveys, three of which were conducted in Germany, one in China, and one among people with Chinese roots living in Germany.
Data source location	City: Beijing, Trier, Magdeburg and other German cities Country: Germany and China
Data accessibility	With the article, future updates may be made available through the webpage of the corresponding author.
Related research article	M. O. Rieger, Triggering Altruism Increases the Willingness to Get Vaccinated Against COVID-19. Social Health and Behavior 3(3) (2020), 78. M. O. Rieger, To wear or not to wear? Factors influencing wearing face masks in Germany during the COVID-19 pandemic. Social Health and Behavior 3(2) (2020) 50-54. M. O. Rieger, What makes young people think positively about social distancing during the Corona crisis in Germany? To appear in: Frontiers of Sociology 5 (2020) 61.

## Value of the Data

• Our data cover a variety of aspects related to the COVID-19 pandemic: worries about the situation, estimations and expectations concerning the further development of the pandemic, perception of government responses and media coverage, attitudes towards social distancing and other countermeasures, and COVID-19-related conspiracy theories.
• The data are valuable for politicians, educators, researchers, media and institutions in public health because they can help to develop ways for improving public attitudes towards the implemented measures and compliance, as well as public trust in government actions.
• The dataset can be used for further insights and for designing experiments by comparing with other similar researches in a meta-analysis.
• The German data were collected at three time points between March and June 2020. During this time period, different measures and restrictions were introduced in Germany. This allows to measure changes in these parameters induced by the changing pandemic situation as well as by changes in policy measures.
• The data also allow making comparisons across countries (China and Germany) that show interesting differences. It should be emphasized, however, that the data are not representative, thus we cannot generalize sample means to the national populations of China or Germany. The primary goal is therefore the detection of associative relationships between the variables.

## 1 Data Description

The attached dataset contains six sub-datasets. Table 1 summarizes the time, place, target group, and the sample characteristics of the six surveys. Collected in the period between March 23 and September 15, the data consist of 1169 responses in total from 865 respondents in Germany (sample 1, wave 1–3.5), 135 in China (sample 2) and 169 with Chinese roots in Germany (sample 3, wave1-2). Of all respondents, females account for, on average, 65%. 61% are University students, and the average age is 27, meaning our survey data are dominated by rather young and educated participants.

**Table 1:** Sample characteristics of the various survey samples and waves. Waves 3 and 3.5 contained the exact same questions.

Samples (waves)	Time	Place/target group	Obser-vation	Female	Univ. students	Mean age	Secondary general school/Middle School/Abitur	University degree
sample1 (wave1)	March 23–26	Trier, Germany	266	66%	82%	26 (14–62)	97%	50%
sample1 (wave2)	April 21–22	Trier, Germany	268	64%	64%	28 (18–77)	97%	46%
sample1 (wave3)	May 17–June 05	Trier, Germany	282	60%	76%	27 (18–65)	97%	49%
sample1 (wave3.5)	June 09–11	Magdeburg, Germany	49	76%	96%	24 (19–36)	100%	49%
sample 2	April 22	Beijing, China	135	72%	96%	22 (18–54)	96%	21%
sample 3 (wave 1)	May 26–June 06	People with Chinese roots in Germany	91	68%	37%	34 (18–60)	89%	78%

sample 3 (wave 2)	August 29–September 15	People with Chinese roots in Germany	78	63%	38%	32(20–71)	91%	83%

Our data were collected through six self-administered anonymous questionnaire-based surveys on the Internet platform Unipark. As shown in Table 1, the respondents of the first sample (wave 1–3) were students and employees of the University of Trier who were informed about the survey through the University email information system, while the data of the wave 3.5, consisting of the same questions as wave 3, were collected through an online survey at the University of Magdeburg, Germany. The data of sample 2 were collected mainly from students of a top university in Beijing, while the data of sample 3 (wave 1–2) were collected through two online surveys advertised mainly on WeChat, the most popular social media platform among people with Chinese roots living in different cities in Germany, including Trier, Magdeburg and Düsseldorf.^1^ As an incentive for participation, a prize of 50 € for one participant each for the three waves of sample 1, and 20 € for ten participants for sample 3 was offered. On average, it took the respondents 10–15 min to finish the questionnaires with an exception of the shorter questionnaire of sample 2 which took them less than 5 min.

The samples in our data are obviously not representative for the overall population of Germany or China. Table 1 summarizes^2^^,^^3^^,^^4^ demographic parameters and Table 2 gives^5^^,^^6^^,^^7^ the corresponding parameters for the total population. Nevertheless, the data can be used for cross-population comparison, for comparisons across time and for studying relations between different survey variables.

**Table 2:** Major demographic benchmarks of target population.

Population	Time	Total	Female	Average age	Secondary general school/Middle School/Abitur	University degree
Germany	31.12.2019	83,166,711	51%	44,5	78%	18%
China	2020	1,439,300,000	47%	37,5	53%	9%
With Chinese citizenship in Germany	31.12.2019	149195	53%	32		

Table 3, Table 4, Table 5, Table 6, Table 7, Table 8 list the variables of the provided dataset which can be used to measure the attitudes of the respondents to various measures introduced to curb the spread of the virus and their perception of government responses. They also include data which can help illustrate the reasons or factors contributing to these attitudes and perceptions such as worries of the participants, experience with infections in their personal environment, their expectations and how informed they are about it, tendency to believe in conspiracy theories and prejudices, critical thinking, as well as their risk and time preferences. The samples and waves listed here show which variables were contained in which surveys. Survey questions can be found in the appendix. The discussion on the theoretical background, analysis of the survey variables and interpretation of the results can be found in related research articles [1,^2^, 3].

**Table 3:** Personal experience, worries and expectations, and degree of awareness about COVID-19.

Variables	Value labels/explanations	Samples (waves)
*Subjects had read about results of previous survey waves:*		
Idfn	Subject identification number variable	1–3 (all)
pre_results	1=yes, 0=no	1 (2, 3, 3.5)
*Personal experiences:*		
known_old_people	number of people over 70 are known to him/her	1 (1, 2, 3, 3.5)
known_case	1=yes, 0=no	1 (1, 2, 3, 3.5)
known_suspected	1=yes, 0=no	1 (1, 2, 3, 3.5)
*Worries and expectations about COVID-19:*		
worries_corona	1=very little, 2=little, 3=somewhat, 4=quite a bit, 5=a great deal	1 (1, 2, 3, 3.5), 3(1, 2)
german_deaths	deaths expected in Germany by the end of 2020	1 (1, 2, 3, 3.5)
chinese_deaths	deaths already reported in China	1 (1, 2)
*Degree of awareness about COVID-19:*		
ways_transmission	1=answer correct, 0=answer wrong	1 (1)
coronavirus_history	1=answer correct, 0=answer wrong	1 (1)
pathogen	1=answer correct, 0=answer wrong	1 (1)
new_influenza_virus	1=answer correct, 0=answer wrong	1 (1)
spread_with_symptom	1=answer correct, 0=answer wrong	1 (1)
mild_illness	1=answer correct, 0=answer wrong	1 (1)
death_rate	1=answer correct, 0=answer wrong	1 (1)
length_vaccine	1=answer correct, 0=answer wrong	1 (1)
exponential_growth	infections expected a month later given the number doubles in four days (correct answer should be around 250,000)	1 (1, 2)

**Table 4:** Critical thinking, risk and time preferences.

Variables	Value labels/explanations	Samples (waves)
*Critical thinking:*		
one_soure_info	1=strongly disagree, 2=disagree, 3=neutral, 4=agree, 5=strongly agree	1 (2, 3, 3.5)
critical_info	1=strongly disagree, 2=disagree, 3=neutral, 4=agree, 5=strongly agree	1 (2, 3, 3.5)
problem_sovling	1=strongly disagree, 2=disagree, 3=agree, 4=strongly agree	1 (3, 3.5), 2
supportive_arguments	1=strongly disagree, 2=disagree, 3=agree, 4=strongly agree	1 (3, 3.5), 2
time_wasting	1=strongly disagree, 2=disagree, 3=agree, 4=strongly agree	1 (3, 3.5), 2
own_thought	1=strongly disagree, 2=disagree, 3=agree, 4=strongly agree	1 (3, 3.5), 2
no_own_ideas	1=strongly disagree, 2=disagree, 3=agree, 4=strongly agree	1 (3, 3.5), 2
*Risk and time preferences:*		
risk_taking	Likert-scale where 1=very high risk aversion, -11=very high risk loving	1 (3, 3.5)
risk_taking_health	Likert-scale where 1=very high risk aversion, -11=very high risk loving	1 (3, 3.5)
patience	1=3400€ this month, 2= 3800€ next month	1 (1)
	1=1700€ this month, 2= 1900€ next month	1 (3, 3.5)

**Table 5:** Opinions on the origin of COVID-19 and tendency to believe in conspiracy theories about COVID-19.

Variables	Value labels/explanations	Samples (waves)
*Opinion on the origin:*		
cov_from_china	1=no, 2=rather unlikely, 3=rather likely, 4=yes	2, 3 (1, 2)
cov_from_usa	1=no, 2=rather unlikely, 3=rather likely, 4=yes	2, 3 (1, 2)
cov_from_elsewhere	1=no, 2=rather unlikely, 3=rather likely, 4=yes	2, 3 (1, 2)
*Subjects have ever heard about the following theories on the origin:*		
animals	1=yes (has heard about it), 0=no (has not heard about it)	1 (2, 3, 3.5)
wuhan_origin	1=yes (has heard about it), 0=no (has not heard about it)	1 (2, 3, 3.5)
wuhan_lab	1=yes (has heard about it), 0=no (has not heard about it)	1 (2, 3, 3.5)
us_secret_service	1=yes (has heard about it), 0=no (has not heard about it)	1 (2, 3, 3.5)
china_bioweapon	1=yes (has heard about it), 0=no (has not heard about it)	1 (2, 3, 3.5)
caused_by_5G	1=yes (has heard about it), 0=no (has not heard about it)	1 (2, 3, 3.5)
pharma_companies	1=yes (has heard about it), 0=no (has not heard about it)	1 (2, 3, 3.5)
wuhan_market	1=yes (has heard about it), 0=no (has not heard about it)	1 (2, 3, 3.5)
*Probability of the theories listed above to be true:*		
prob_animals	1=very unlikely, 2=unlikely, 3=somewhat likely, 4=likely, 5=very likely	1 (2, 3, 3.5), 3 (1)
prob_wuhan_origin	1=very unlikely, 2=unlikely, 3=somewhat likely, 4=likely, 5=very likely	1 (2, 3, 3.5), 3 (1, 2)
prob_wuhan_lab	1=very unlikely, 2=unlikely, 3=somewhat likely, 4=likely, 5=very likely	1 (2, 3, 3.5), 3 (1)
prob_US	1=very unlikely, 2=unlikely, 3=somewhat likely, 4=likely, 5=very likely	1 (2, 3, 3.5), 3 (1, 2)
prob_China_bioweapon	1=very unlikely, 2=unlikely, 3=somewhat likely, 4=likely, 5=very likely	1 (2, 3, 3.5), 3 (1)
prob_5G	1=very unlikely, 2=unlikely, 3=somewhat likely, 4=likely, 5=very likely	1 (2, 3, 3.5), 3 (1)
prob_pharma_companies	1=very unlikely, 2=unlikely, 3=somewhat likely, 4=likely, 5=very likely	1 (2, 3, 3.5), 3 (1)
prob_wuhan_market	1=very unlikely, 2=unlikely, 3=somewhat likely, 4=likely, 5=very likely	1 (2, 3, 3.5), 3 (1)
*Tendency to believe in conspiracy theories:*		
trust_offi_info	1=disagree, 2=somewhat agree, 3=mostly agree, 4=strongly agree	1 (1, 2, 3, 3.5), 3 (1)
media_hide_info	1=disagree, 2=somewhat agree, 3=mostly agree, 4=strongly agree	1 (1, 2, 3, 3.5)
german_media_hide_info	1=disagree, 2=somewhat agree, 3=mostly agree, 4=strongly agree	3 (1)
chinese_media_hide_info	1=disagree, 2=somewhat agree, 3=mostly agree, 4=strongly agree	2, 3 (1)
profit_pharma	1=disagree, 2=somewhat agree, 3=mostly agree, 4=strongly agree	1 (1, 2, 3, 3.5), 2, 3 (1)
rights_undermining	1=disagree, 2=somewhat agree, 3=mostly agree, 4=strongly agree	1 (2, 3, 3.5), 3 (1)

**Table 6:** Prejudices during COVID-19.

Variables	Value labels/explanations	Samples (waves)
avoid_italian	1=disagree, 2=somewhat agree, 3=mostly agree, 4=strongly agree	1 (2)
avoid_french	1=disagree, 2=somewhat agree, 3=mostly agree, 4=strongly agree	1 (2)
avoid_chinese	1=disagree, 2=somewhat agree, 3=mostly agree, 4=strongly agree	1 (1, 2)
chinese_responsible	1=disagree, 2=somewhat agree, 3=mostly agree, 4=strongly agree	1 (1, 2, 3, 3.5)
econ_relations_reduction	1=disagree, 2=somewhat agree, 3=mostly agree, 4=strongly agree	1 (1, 2, 3, 3.5)
less_chinese	1=disagree, 2=somewhat agree, 3=mostly agree, 4=strongly agree	1 (1, 2, 3, 3.5)
no_globalization	1=disagree, 2=somewhat agree, 3=mostly agree, 4=strongly agree	1 (1, 2, 3, 3.5), 3 (1)

Table 3 contains questions on personal experiences, worries and expectations, and the degree of awareness about COVID-19 of the subjects. Participants were asked whether they had read about the results of previous survey waves and about their personal experience, including the number of people over 70 they know (a typical risk group for COVID-19) and whether they know anyone with a confirmed or suspected case of COVID-19. Questions on the degree of their worries about COVID-19, the number of COVID-19 related deaths they expect to occur in Germany until the end of 2020, and deaths from COVID-19 already reported in China (according to official figures) were also included. To measure how well-informed they were about the virus and the disease, a total of nine statements including the ways of transmission of the virus, the mortality rate and exponential growth were provided. For the first eight items which comprise both correct and wrong statements, respondents had to mark them as correct or not. The sum of the correct answers is taken to measure their degree of awareness about the virus.

To see whether they are aware of the exponential growth of cases during a pandemic, the respondents were asked to estimate the number of cases in one month, assuming that the numbers double in four days.

Questions in Table 4 would help measure critical thinking, risk and time preferences of the participants. To measure critical thinking, they were asked whether they were satisfied with one single source when searching for information about a specific topic or whether they searched for information both confirming and contradicting their opinion and weigh the arguments against one another. Aside from these, they were asked to state to what extent they agree or disagree with the following five statements which based on [4]: “It is not very important to insist on trying to solve a difficult problem”; “I search arguments that support my point of view and I do not search any counterarguments”; “Analysing the arguments of others is a waste of time”; “I am aware of my own thoughts, so why should I pretend to be thinking about other options?”; “Taking into account other people's ideas means that you cannot have your own”. By asking to assess their risk preferences in general and in relation to their health, we want to assess their risk preference. These two questions are from SOEP^8^ and have been tested regarding their reliability in [5]. To find out their time preference or patience, they were given two choices and they could select one of them: either 3400€ (1700€ for sample 1, wave 3 and 3.5) this month or 3800€ (1900€ for sample 1, wave 3 and 3.5) next month. This question was adapted from [6] and has also been used in this form in [7].

Table 5 consists of questions on the opinion of the respondents on the origin of COVID-19 and questions to measure the tendency to believe in COVID-19-related conspiracy theories. As to the opinion on the origin of the virus, the respondents were asked to assess how likely it was that the virus originated in China, the USA or elsewhere. Next, a series of theories on the origin of COVID-19 were listed. These include not only statements with scientific consensus such as that the virus originated in animals and in Wuhan (China), but also popular conspiracy theories such as that the virus was developed by the US secret service or at a Chinese laboratory for bio-weapons. Participants were asked whether they had ever heard about each of these theories and then to assess the probability of each of these theories being true. Furthermore, we also less directly measured how likely the respondents were to believe in conspiracy theories by asking the following four questions: whether they trust official information on the virus, whether the media (more specifically in sample 2 and 3 (wave 1): German or Chinese media) try to hide relevant information, whether the “hype” about corona is just caused by pharmaceutical companies, and whether politicians just want to make use of the chance to undermine people's fundamental rights.

To measure the attitude towards foreigners (Chinese, French and Italians, as these were at the given time and location most associated with COVID-19) and globalization, participants were asked to state whether they agree with the following statements (Table 6): “I could understand when someone would avoid sitting next to an Italian/French/Chinese person while on the bus”; “Chinese are responsible for the pandemic”; “We should reduce the scale of economic relations with China to avoid such problems in the future”; “It would be better if there were less Chinese people in Germany”; “Our life would be better without globalization”.

Table 7 presents the questions that aim to assess the attitude towards the measures taken in the fight against COVID-19. On the one hand, subjects were asked whether they agreed that China's response to the outbreak was better than that of Germany and that Germany should learn from East-Asia how to deal with pandemics in general, or whether they agreed that Wuhan was too slow in its response to the pandemic. On the other hand, specific questions regarding social distancing and face masks were included. As regards social distancing, five hypothetical scenarios involving different behaviours of university students were presented: A student celebrates his birthday with his friends while none of them belongs to a risk-group for coronavirus; A student plays soccer with his with friends while none of them shows symptoms of a cold; A student visits her lonely grandma in a nursing home despite the fact that she has a cold; A student refuses to hug his friends as usual and insists on keeping distance when they meet, even though none of them is sick; A student tells a friend that it is irresponsible of him to continue to meet his friends, knowing that it may hurt the friend's feelings. These five scenarios were listed in a randomized order and respondents could rate their attitude toward each scenario on a scale of “1=perfectly OK” to “4=unacceptable”. To measure their attitude toward social distancing, the average of scenarios 4 and 5 was subtracted from the average result of the first three scenarios. Next, the respondents were asked to estimate the duration of the restriction (lockdown).

**Table 7:** Attitudes toward social distancing and face masks.

Variables	Value labels/explanations	Samples (waves)
*Countermeasures general:*		
china_better	1=disagree, 2=somewhat agree, 3=mostly agree, 4=strongly agree	1 (1, 2, 3, 3.5), 3 (1)
lern_eastasia	1=disagree, 2=somewhat agree, 3=mostly agree, 4=strongly agree	1 (2, 3, 3.5)
wuhan_response	1=disagree, 2=somewhat agree, 3=mostly agree, 4=strongly agree	3 (1)
*Social distancing:*		
hypo_birthday_party	1=perfectly OK, 2=not optimal but understandable, 3=rather bad, 4=unacceptable	1 (1, 3, 3.5)
hypo_soccer	1=perfectly OK, 2=not optimal but understandable, 3=rather bad, 4=unacceptable	1 (1, 3, 3.5)
hypo_grandma_visiting	1=perfectly OK, 2=not optimal but understandable, 3=rather bad, 4=unacceptable	1 (1, 3, 3.5)
hypo_distance_keeping	1=perfectly OK, 2=not optimal but understandable, 3=rather bad, 4=unacceptable	1 (1, 3, 3.5)
hypo_blaming	1=perfectly OK, 2=not optimal but understandable, 3=rather bad, 4=unacceptable	1 (1, 3, 3.5)
estimated_restriction_duration	in weeks	1 (1)
*Opinion on face masks:*		
weird_masks	1=disagree, 2=somewhat agree, 3=mostly agree, 4=strongly agree	1 (1, 2, 3, 3.5)
seen_as_weird	1=yes, 2=rather yes, 3=rather no, 4=no	1 (2, 3, 3.5)
self_protection	1=very well, 2=well, 3=somewhat, 4= very little, 5=not at all	1 (2, 3, 3.5)
protection_others	1=very well, 2=well, 3=somewhat, 4= very little, 5=not at all	1 (2, 3, 3.5)
*Whether they would voluntarily wear a face mask at the following locations:*		
mask_supermarket	1=no, 2=probably no, 3=probably yes, 4=yes	1 (2, 3, 3.5)
mask_bus	1=no, 2=probably no, 3=probably yes, 4=yes	1 (2, 3, 3.5)
mask_university	1=no, 2=probably no, 3=probably yes, 4=yes	1 (2, 3, 3.5)
mask_street	1=no, 2=probably no, 3=probably yes, 4=yes	1 (2, 3, 3.5)
mask_plane	1=no, 2=probably no, 3=probably yes, 4=yes	1 (2)
*Whether they would wear a face mask at the following locations if legally required:*		
law_mask_supermaket	1=no, 2=probably no, 3=probably yes, 4=yes	1 (2)
law_mask_bus	1=no, 2=probably no, 3=probably yes, 4=yes	1 (2)
law_mask_university	1=no, 2=probably no, 3=probably yes, 4=yes	1 (2)
law_mask_street	1=no, 2=probably no, 3=probably yes, 4=yes	1 (2)
law_mask_plane	1=no, 2=probably no, 3=probably yes, 4=yes	1 (2)

As regards face masks, the questions can be divided into three groups. In the first group, the general opinion of the respondents on face masks was asked: whether it is weird when someone wears a mask in public; whether others would think strange of them if they wear a mask; how well wearing a face mask can protect oneself from getting infected; how well it can protect others from getting infected. In the second part, they were asked whether they would wear a face mask voluntarily when they were at a supermarket, on a bus, at the University, on the street and on a plain, respectively. Too see whether they would change their decisions when face mask wearing was a legal obligation, the same questions were asked again in the third part but under the condition that it were required by law.

Last but not least, to assess the participants’ satisfaction with the government performance during the pandemic, they were asked, as listed in Table 8, to state how satisfied they were on a scale from 1 to 7 with the provision of information on the virus, measures taken against the pandemic, and measures to ensure the normal course of daily life of the citizens during the pandemic. Furthermore, they were also asked to rate the response of the German, Chinese and Wuhan governments to the Coronavirus in general. The possible answers ranged from “1=far too slow/lax” to “5=far too fast/restrictive”.

**Table 8:** Perceptions of government reactions.

Variables	Value labels/explanations	Samples (waves)
gov_info_provision	Likert-scale where 1=very dissatisfied, -7=very satisfied	1 (1, 2, 3, 3.5)
gov_measures_pandemic	Likert-scale where 1=very dissatisfied, -7=very satisfied	1 (1, 2, 3, 3.5)
gov_measures_daily_life	Likert-scale where 1=very dissatisfied, -7=very satisfied	1 (1, 2, 3, 3.5)
germany_gov_slow_fast	1=far too slow and lax, 2=rather slow and lax, 3=well-balanced, 4=rather fast and restrictive, 5=far too fast and restrictive	1 (1, 2, 3, 3.5), 3 (1, 2)
china_gov_slow_fast	1=far too slow and lax, 2=rather slow and lax, 3=well-balanced, 4=rather fast and restrictive, 5=far too fast and restrictive	3 (2)
wuhan_ gov_slow_fast	1=far too slow and lax, 2=rather slow and lax, 3=well-balanced, 4=rather fast and restrictive, 5=far too fast and restrictive	3 (2)

## 2 Experimental Design, Materials and Methods

There was one questionnaire for each wave of sample 1 and sample 3, and one for sample 2 respectively. Each questionnaire consisted of two parts. The first part included general information on the respondents (gender, age, job, highest level of education) which is summarized in Table 1. The second part consisted, as mentioned above, of questions on worries, personal experience, expectations and degree of awareness of about the virus, tendency to believe in conspiracy theories, prejudices during COVID-19, critical thinking, as well as risk and time preferences, but also included questions on the opinion of the respondents on the measures taken and the perception of government responses. This part was adapted for each survey sample.

## Survey Questions

(age) What is your age?

(gender) What is your gender?

1. Male
2. Female
3. Other

(job) What is your current occupation?

1. Pupil
2. Student
3. Employed (public sector)
4. Employed (private sector)
5. Self-employed
6. Homemaker
7. Unemployed
8. Other

(degree) What is the highest degree or level of school you have completed?

1. Secondary general school (Hauptschule)/Middle School (Mittlere Reife)/Abitur
2. Bachelor's degree
3. Master's degree, Magister degree, Diploma
4. PhD
5. Other

(econ_experience) Are you currently pursuing or do you already have a degree in Economics?

1. Yes
2. No

(pre_results) Have you had the opportunity to get acquainted with the results of our previous survey on the Coronavirus crisis?

1. Yes
2. No

(known_old_people) How many people aged 70 or above do you personally know?

(known_case) Did any of your relatives, acquaintances or friends get infected with the novel Coronavirus?

1. Yes
2. No

(known_suspected) Does any of your relatives, acquaintances or friends suspect to have been infected with the novel Coronavirus?

1. Yes
2. No

(worries_corona) How worried are you personally about the consequences of the Coronavirus?

1. Very little
2. Little
3. Somewhat
4. Quite a bit
5. A great deal

(german_deaths) How many people do you think will die from Coronavirus in Germany until the end of this year?

(chinese_deaths) How many people do you think have died from Coronavirus in China until now (according to official figures)?

Which of the following statements do you think are correct?

(ways_transmission) Coronaviruses can be spread when the infected person coughs, sneezes but also speaks or breathes.

(coronavirus_history) Coronaviruses have always been there and mostly cause a harmless cold.

(pathogen) The correct name of the ongoing virus is SARS-CoV-19.

(new_influenza_virus) Coronavirus is a new form of the flu virus.

(spread_with_symptom) Only those with symptoms can spread the virus.

(mild_illness) In most cases, the virus leads to a mild cold.

(death_rate) The mortality rate of the virus is 0.1% among young people and in the double-digit percent range among older people.

(length_vaccine) According to the experts, the development of a vaccine will take about a year.

(exponential_growth) If the number of infected people in a given country is 1000 on March 20 and 1000 new cases are reported on March 24, how many new cases can be expected in a month (given that no measures are being undertaken to curb the spread of the virus).

To what extent do you agree or disagree with the following statements?

(one_soure_info) While searching for new information on a specific topic, I am normally satisfied with one single source.

1. Strongly disagree
2. Somewhat disagree
3. Neither agree nor disagree
4. Somewhat agree
5. Strongly agree

(critical_info) While searching for new information, I search both for information that supports my opinion as well as for information that contradicts my opinion and I weigh the arguments against one another.

1. Strongly disagree
2. Somewhat disagree
3. Neither agree nor disagree
4. Somewhat agree
5. Strongly agree

(problem_sovling) It is not very important to insist on trying to solve a difficult problem.

1. Strongly disagree
2. Disagree
3. Agree
4. Strongly agree

(supportive_arguments) I search for arguments that support my point of view and not for arguments that contradict it.

1. Strongly disagree
2. Disagree
3. Agree
4. Strongly agree

(time_wasting) Analyzing the arguments of other people is a waste of time.

1. Strongly disagree
2. Disagree
3. Agree
4. Strongly agree

(own_thought) I am aware of my own opinions, why should I pretend to be considering other options?

1. Strongly disagree
2. Disagree
3. Agree
4. Strongly agree

(no_own_ideas) Taking into account the opinions of other people means that you are not able to have your own.

1. Strongly disagree
2. Disagree
3. Agree
4. Strongly agree

(risk_taking) How would you personally assess yourself? Are you generally willing to take risks or do you try to avoid taking risks?

1. 0=very high risk aversion
2. 1
3. 2
4. 3
5. 4
6. 5
7. 6
8. 7
9. 8
10. 9
11. 10=very high risk loving

(risk_taking_health) How would assess your willingness to take risks in situations related to your health?

1. 0=very high risk aversion
2. 1
3. 2
4. 3
5. 4
6. 5
7. 6
8. 7
9. 8
10. 9
11. 10=very high risk loving

(patience) Which of the following offers would you prefer?

1. A payment of 3400 € this month
2. A payment of 3800 € next month

(patience) Which of the following offers would you prefer?

1. A payment of 1700 € this month
2. A payment of 1900 € next month

Where do you think does the COVID-19 origin from?

(cov_from_china) China

1. No
2. Rather unlikely
3. Rather likely
4. Yes

(cov_from_usa) The USA

1. No
2. Rather unlikely
3. Rather likely
4. Yes

(cov_from_elsewhere) Elsewhere

1. No
2. Rather unlikely
3. Rather likely
4. Yes

Have you heard or read any of the following theories about the origins of COVID-19?

(animals) The virus originated in animals (bats or pangolins) and spread to humans.

1. Yes, I have.
2. No, never.

(wuhan_origin) The virus emanated in Wuhan (China).

1. Yes, I have.
2. No, never.

(wuhan_lab) The first patient was an employee of a virus lab in Wuhan who got infected by accident.

1. Yes, I have.
2. No, never.

(us_secret_service) The US Secret Agency developed the virus and imported it into Wuhan to damage China.

1. Yes, I have.
2. No, never.

(china_bioweapon) The virus was developed by China at a laboratory for biological weapons and spread due to an accident.

1. Yes, I have.
2. No, never.

(caused_by_5G) The spread of COVID-19 is related to the rollout of 5G networks.

1. Yes, I have.
2. No, never.

(pharma_companies) Pharmaceutical companies and Bill Gates spread the virus to make money from their patented vaccine.

1. Yes, I have.
2. No, never.

(wuhan_market) The first patients supposedly got infected at a live animal market in Wuhan.

1. Yes, I have.
2. No, never.

How likely do you think these theories about the origins of COVID-19 are?

(prob_animals) The virus originated in animals (bats or pangolins) and spread to humans.

1. Very unlikely
2. Unlikely
3. Somewhat likely
4. Likely
5. Very likely

(prob_wuhan_origin) The virus emanated in Wuhan (China).

1. Very unlikely
2. Unlikely
3. Somewhat likely
4. Likely
5. Very likely

(prob_wuhan_lab) The first patient was an employee of a virus lab in Wuhan who got infected by accident.

1. Very unlikely
2. Unlikely
3. Somewhat likely
4. Likely
5. Very likely

(prob_US) The US Secret Agency developed the virus and imported it into Wuhan to damage China.

1. Very unlikely
2. Unlikely
3. Somewhat likely
4. Likely
5. Very likely

(prob_China_bioweapon) The virus was developed by China at a laboratory for biological weapons and spread due to an accident.

1. Very unlikely
2. Unlikely
3. Somewhat likely
4. Likely
5. Very likely

(prob_5G) The spread of COVID-19 is related to the rollout of 5G networks.

1. Very unlikely
2. Unlikely
3. Somewhat likely
4. Likely
5. Very likely

(prob_pharma_companies) Pharmaceutical companies and Bill Gates spread the virus to make money from a patented vaccine developed by them.

1. Very unlikely
2. Unlikely
3. Somewhat likely
4. Likely
5. Very likely

(prob_wuhan_market) The first patients supposedly got infected at a live animal market in Wuhan.

1. Very unlikely
2. Unlikely
3. Somewhat likely
4. Likely
5. Very likely

To what extent do you agree or disagree with the following statements?

(trust_offi_info) I trust the official information on the Coronavirus in Germany.

1. Disagree
2. Somewhat agree
3. Mostly agree
4. Strongly agree

(media_hide_info) The media try to hide information about the Coronavirus from the public.

1. Disagree
2. Somewhat agree
3. Mostly agree
4. Strongly agree

(german_media_hide_info) The German media try to hide information about the Coronavirus from the public.

1. Disagree
2. Somewhat agree
3. Mostly agree
4. Strongly agree

(chinese_media_hide_info) The Chinese media try to hide information about the Coronavirus from the public.

1. Disagree
2. Somewhat agree
3. Mostly agree
4. Strongly agree

(profit_pharma) The hype about the Coronavirus was caused by pharmaceutical companies and other groups that benefit from it.

1. Disagree
2. Somewhat agree
3. Mostly agree
4. Strongly agree

(rights_undermining) The virus is just an excuse for our politicians to trample on our fundamental human rights.

1. Disagree
2. Somewhat agree
3. Mostly agree
4. Strongly agree

(avoid_italian) I can understand why someone would avoid sitting next to an Italian person on the bus.

1. Disagree
2. Somewhat agree
3. Mostly agree
4. Strongly agree

(avoid_french) I can understand why someone would avoid sitting next to a French person on the bus.

1. Disagree
2. Somewhat agree
3. Mostly agree
4. Strongly agree

(avoid_chinese) I can understand why someone would avoid sitting next to a Chinese person on the bus.

1. Disagree
2. Somewhat agree
3. Mostly agree
4. Strongly agree

(chinese_responsible) Ultimately, it is the Chinese that are responsible for the pandemic.

1. Disagree
2. Somewhat agree
3. Mostly agree
4. Strongly agree

(econ_relations_reduction) We should limit our economic relations with China to avoid such problems in the future.

1. Disagree
2. Somewhat agree
3. Mostly agree
4. Strongly agree

(less_chinese) It would be better if there were less Chinese people in Germany.

1. Disagree
2. Somewhat agree
3. Mostly agree
4. Strongly agree

(no_globalization) Our lives would be better without globalization.

1. Disagree
2. Somewhat agree
3. Mostly agree
4. Strongly agree

(china_better) China's response to the pandemic was better than that of Germany.

1. Disagree
2. Somewhat agree
3. Mostly agree
4. Strongly agree

(lern_eastasia) We can generally learn from East Asia how to handle pandemics.

1. Disagree
2. Somewhat agree
3. Mostly agree
4. Strongly agree

(wuhan_response) Wuhan was too slow in its response to the pandemic.

1. Disagree
2. Somewhat agree
3. Mostly agree
4. Strongly agree

How would you characterize the behavior of the following students last weekend?

(hypo_birthday_party) A student celebrates his birthday with his friends on the university campus. None of the friends is in a high-risk group.

1. Perfectly OK
2. Not optimal but understandable
3. Rather bad
4. Unacceptable

(hypo_soccer) A student meets his friends for a soccer game. None of them is showing any symptoms.

1. Perfectly OK
2. Not optimal but understandable
3. Rather bad
4. Unacceptable

(hypo_grandma_visiting) Despite having a cold, a student visits his grandmother in a nursing home because she is feeling lonely.

1. Perfectly OK
2. Not optimal but understandable
3. Rather bad
4. Unacceptable

(hypo_distance_keeping) A student meets his friends for a jog and does not greet them by hugging as usual and insists that they keep distance, even though his friends tell him that they are not feeling sick.

1. Perfectly OK
2. Not optimal but understandable
3. Rather bad
4. Unacceptable

(hypo_blaming) A student tells her friend that it is irresponsible of him to continue to meet his friends, knowing that it may hurt his feelings.

1. Perfectly OK
2. Not optimal but understandable
3. Rather bad
4. Unacceptable

(estimated_restriction_duration) How many weeks do you think the ongoing restrictions due to the Coronavirus will last in Germany?

(weird_masks) It is weird when someone wears a face mask in public.

1. Disagree
2. Somewhat agree
3. Mostly agree
4. Strongly agree

(seen_as_weird) Would others (friends, colleagues, etc.) think strange of you if you wore a face mask?

1. Yes, for sure
2. Rather yes
3. Rather no
4. No, certainly not

(self_protection) How well do you think wearing a face mask protects one from getting infected with the new Coronavirus?

1. Very well
2. Well
3. Somewhat
4. Very little
5. Not at all

(protection_others) How well do you think wearing a face mask prevents one from infecting others with the new Coronavirus?

1. Very well
2. Well
3. Somewhat
4. Very little
5. Not at all

Would you wear a face mask in the following situations (assuming you had one)?

(mask_supermarket) At the supermarket

1. No
2. Probably no
3. Probably yes
4. Yes

(mask_bus) On the bus

1. No
2. Probably no
3. Probably yes
4. Yes

(mask_university) At the university

1. No
2. Probably no
3. Probably yes
4. Yes

(mask_street) On the street

1. No
2. Probably no
3. Probably yes
4. Yes

(mask_plane) On the plane

1. No
2. Probably no
3. Probably yes
4. Yes

If wearing a face mask were legally required, would you wear one in the following situations (assuming you had one)?

(law_mask_supermaket) At the supermarket

1. No
2. Probably no
3. Probably yes
4. Yes

(law_mask_bus) On the bus

1. No
2. Probably no
3. Probably yes
4. Yes

(law_mask_university) At the university

1. No
2. Probably no
3. Probably yes
4. Yes

(law_mask_street) On the street

1. No
2. Probably no
3. Probably yes
4. Yes

(law_mask_plane) On the plane

1. No
2. Probably no
3. Probably yes
4. Yes

How satisfied are you with the performance of the Federal Government on the following aspects?

(gov_info_provision) Provision of information on the novel Coronavirus

1. 1=very dissatisfied
2. 2
3. 3
4. 4
5. 5
6. 6
7. 7=very satisfied

(gov_measures_pandemic) Measures aimed at managing the Coronavirus crisis (e.g. timely provision of hospital beds)

1. 1=very dissatisfied
2. 2
3. 3
4. 4
5. 5
6. 6
7. 7=very satisfied

(gov_measures_daily_life) Measures that ensure the normal course of daily life of the citizens during the ongoing pandemic (e.g. ensuring the availability of essential items)

1. 1=very dissatisfied
2. 2
3. 3
4. 4
5. 5
6. 6
7. 7=very satisfied

(germany_gov_slow_fast) All in all, how would you rate the response of the German government to the novel Coronavirus?

1. Far too slow and lax
2. Rather slow and lax
3. Well-balanced
4. Rather fast and restrictive
5. Far too fast and restrictive

(china_gov_slow_fast) All in all, how would you rate the response of the Chinese government to the novel Coronavirus?

1. Far too slow and lax
2. Rather slow and lax
3. Well-balanced
4. Rather fast and restrictive
5. Far too fast and restrictive

(wuhan_gov_slow_fast) All in all, how would you rate the response of the Wuhan government to the novel Coronavirus?

1. Far too slow and lax
2. Rather slow and lax
3. Well-balanced
4. Rather fast and restrictive
5. Far too fast and restrictive

## Ethics Statement

Informed consent was obtained for experimentation with human subjects.

## Declaration of Competing Interest

The authors declare that they have no known competing financial interests or personal relationships which have, or could be perceived to have, influenced the work reported in this article.

## Appendix Supplementary materials

Image, application 1

## Footnotes

1 We did not elicit explicitly the nationality or cultural identity of participants, but we know from previous studies with the same sampling method that only around 9% of subjects had a nationality different than German.

2 Obvious typos have not been considered in the statistics.

3 See note 2 above.

4 Different to other samples, more respondents in sample 3 (around 41% in wave 1 and 44% in wave 2) have obtained a master´s degree.

5 Data time point is 2018.

6 Data time point is 31.12.2017.

7 According to the sixth national census 2010, the same for university degree of the population in China.

8 German Socio-Economic Panel, for more information please see here: https://www.diw.de/soep.
